# Independent mobility on the journey to school: A joint cross-sectional and prospective exploration of social and physical environmental influences

**DOI:** 10.1016/j.jth.2013.12.003

**Published:** 2014-03

**Authors:** Alison Carver, Jenna R. Panter, Andrew P. Jones, Esther M.F. van Sluijs

**Affiliations:** a Centre for Physical Activity and Nutrition Research, School of Exercise and Nutrition Sciences, Deakin University, Victoria, Australia; b MRC Epidemiology Unit, Cambridge, UK; c Centre for Diet and Activity Research, University of Cambridge, UK; d Norwich Medical School, University of East Anglia, Norwich, UK

**Keywords:** Active transport, Child, Independent mobility, Built environment, Neighborhood

## Abstract

**Background:**

Despite related physical/mental health benefits, children′s independent mobility for school travel (i.e. walking/cycling without adult accompaniment) has declined in recent decades.

**Purpose:**

To examine cross-sectional and longitudinal associations between social/physical environmental variables and independent mobility on the school journey.

**Methods:**

Participants were 1121 9–10 year-olds residing within 1600 m of their school in urban/rural areas of Norfolk, UK in 2007 (T1). At one year (T2) 491 children were followed-up. At T1, parents survey-reported perceptions of the social/physical environment and rules regarding their child′s physical activity. Characteristics of the neighborhood, route to school and school environment were measured using a Geographical Information System and school audits. At both time-points children survey-reported their usual travel mode and whether accompanied. Regression analyses were conducted in 2013.

**Results:**

Around half walked/cycled to school without adult accompaniment (T1, 43%; T2, 53%). Parents often allowing their child to play outside anywhere within the neighborhood (adjusted odds ratio (AOR) 3.14 (95% CI 1.24–7.96)) and household car access (AOR 0.27 (95% CI 0.08–0.94)) were associated longitudinally with boys walking/cycling independently to school. Land use mix (AOR 1.38 (95% CI 1.06–1.79)), proportion of main roads in the neighborhood (AOR 0.67 (95% CI 0.47–0.94)) and parental encouragement for walking/cycling (AOR 0.40 (95% CI 0.20–0.80)) were associated longitudinally with girls walking/cycling independently to school.

**Conclusions:**

Interventions should develop parents′ skills to teach their children to be independently mobile and to build confidence regarding venturing out without parental accompaniment. Urban planners should consider designing neighborhoods in which residences, business/retail outlets and sports facilities are co-located to promote active transport.

## Highlights

• This novel longitudinal study explored children′s independent mobility to school.
• Being allowed to play outside was related to boys′ independent mobility to school.
• Land use mix was related to girls′ independent mobility to school.
• Interventions should build confidence for children to venture out without adults.
• Urban planners should include mixed land use in neighborhood design.

## 1 Introduction

Having identified physical inactivity as a major risk factor for global mortality, the World Health Organisation (WHO, 2010) recommends that school-aged children engage in at least one hour of moderate-to-vigorous physical activity daily. Nowadays, children′s leisure time is increasingly spent indoors, often using electronic entertainment media (Karsten, 2005, Tandy, 1999). Therefore, strategies that promote physical activity among youth are vital. One example is the promotion of active transport (e.g. walking/cycling) to school, as this may encourage habitual physical activity among school-aged children (Tudor-Locke et al., 2001). Two comprehensive literature reviews of predominantly cross-sectional studies identified that children who walk/cycle to school are more physically active overall than those who travel by motorized modes (Faulkner et al., 2009), and that this behavior is associated with lean body composition and improved cardiorespiratory fitness (Lubans et al., 2011).

Independent mobility refers to children′s freedom to move around their neighborhood (or similar) without adult accompaniment (Hillman et al., 1990). Independent mobility whilst walking/cycling to destinations within the local neighborhood (e.g. school, shops) provides unsupervised opportunities for interaction with the built (Rissotto and Tonucci, 2002) and natural environments (Bixler et al., 2002) and for development of spatial and navigational skills (Rissotto and Tonucci, 2002). It may be beneficial also for building friendships (Prezza et al., 2001) and a sense of community (Prezza and Pacilli, 2007). Despite these benefits children′s independent mobility for school travel has declined in recent decades. For example, a study of English schoolchildren (Hillman et al., 1990) reported that between 1971 and 1990 the proportion of seven-year-olds who traveled to school without adult accompaniment declined from more than 70% to less than 10%. A recent Australian study (Carver et al., 2012) reported low rates (<40%) of walking and cycling to school without adult accompaniment among primary (elementary) schoolchildren in urban areas, and even lower rates (<30%) in rural areas.

According to socio-ecological modeling of health behaviors (Sallis and Owen, 1997) there are multiple layers of influence of intrapersonal, social and physical environment variables on an individual′s health behaviors. In line with the ecological model, it appears that many children now have limited opportunities to spend time outdoors independently due to parental concern about neighborhood safety. Previous research suggests that social environmental factors such as parental concerns about road safety and ‘stranger danger’ are associated with lower levels of independent mobility on the school commute (Hillman et al., 1990, Carver et al., 2008). However there is a paucity of longitudinal research that examines environmental predictors of change in independent mobility. Following the rationale of Moudon and Lee (2003), physical environmental variables that may influence independent mobility on the school journey are the neighborhood surrounding the child′s home, the route to school, and the school environment. However the relative importance of these environments is not known. Hence, the aim of this study was to examine cross-sectional and longitudinal associations between a range of social and physical environmental variables and independent mobility on the journey to school among primary (i.e. elementary) schoolchildren in Norfolk, UK.

## 2 Methods

### 2.1 Sample

Children′s data were drawn from the Sport, Physical activity and Eating behavior: Environmental Determinants in Young people (SPEEDY) study and the recruitment methods have been published previously (van Sluijs et al., 2008, Corder et al., 2010). Briefly, at baseline (T1) 2064 children (response rate 57%) aged 9–10 years were recruited from 92 schools in urban and rural areas of Norfolk, UK. Between April and July 2007 children completed a questionnaire at school, and parents completed a questionnaire distributed in take-home packages. Children were followed up by postal communication at one year (T2) between April and July 2008 (*n*=1019; response rate, 49%), when they completed a second questionnaire while still at the same school. No follow-up information was collected from the parents. There were no significant differences in baseline physical activity levels (Corder et al., 2010) or travel mode between participants and non-participants of the follow-up study. At each time-point, parental consent was obtained prior to data collection. Ethical approval was obtained from the University of East Anglia research ethics committee.

### 2.2 Measures

#### 2.2.1 Independent mobility on the school journey

At each time-point children reported how they usually traveled to school (response options were: by car; bus/train; bicycle; or on foot) and with whom (possible responses were: alone; with a brother/sister; a parent/other adult; a friend; another person – children were asked to select all that applied). At each time-point a dichotomous measure of independent mobility was defined to indicate whether children walked/cycled without adult accompaniment and assigned values of (0) ‘walked/cycled to school with adult accompaniment or used a motorized travel mode’; (1) ‘no adult accompaniment when walking/cycling to school’. Because the likelihood of using active transport decreases sharply with increasing distance, there was examination only of those children who could feasibly walk/cycle to school independently, defined as those residing within 1600 m of their school (Timperio et al., 2004). Although independent mobility was measured at each time-point, explanatory variables were measured only at T1.

#### 2.2.2 Socio-demographic variables

Parents reported car ownership and their highest education level (a proxy for socioeconomic position). This was collapsed into three categories: low (high school leaving certificate or less); medium (vocational above high school); high (university education or above). Children reported their sex, age, number of siblings and bicycle ownership.

#### 2.2.3 Objective environmental measures

Using a Geographical Information System (ESRI ArcGIS 9.2) and the Ordnance Survey database Mastermap Address Layer 2, children′s home addresses were mapped. Area-level socioeconomic deprivation was measured using the English Index of Multiple Deprivation (IMD; Department of Communities and Local Government, 2007) based on these addresses. Objective environmental measures that were significantly associated with active transport to school among these children at T1 (Panter et al., 2010a) were examined as predictors of independent mobility on active school journeys at each time-point. These measures are described previously (Panter et al., 2010b), but briefly are categorized as ‘neighborhood’, ‘route (to school)’, and ‘school’ characteristics (Table 1). Each child′s neighborhood was defined as the area within an 800 m pedestrian network buffer around their home, representing an area within 10 min walk. Characteristics of routes to schools were examined within a 100 m buffer of the shortest route between home and school. School characteristics were identified by an on-foot audit of facilities in the school grounds that may promote walking/cycling and by surveying principals on their school policies towards active transport (Panter et al., 2010a).

**Table 1:** Objective environmental measures for children who reside within 1600 m of their school.

**Characteristic**	**Description**	**Values**
**Neighborhood**^a^**characteristics**		
Road density (mean (SD))	Total road lengths divided by neighborhood area	9.5 (3.9)
Proportion of primary roads (%; median (range))	Length of primary roads divided by total road length	0 (0–43)
Effective walkable area (mean (SD))	Total neighborhood area (the area that can be reached via the street network within 800 m from home) divided by the potential walkable area (a circular buffer with radius 800 m around home)	0.40 (0.13)
Connected node ratio (mean (SD))	Number of junctions divided by number of junctions and cul-de-sacs	0.69 (0.09)
Junction density (mean (SD))	Number of junctions divided by total neighborhood area	6.45 (2.19)
Land-use mix (mean (SD))	Proportion of each land use^b^ squared and summed	2637 (1008)
Socioeconomic deprivation (mean (SD))^c^	Population weighted scores for neighborhood	16.76 (14.67)
Urban/rural status (%)	Urban/rural classification of child′s home address	
Urban		79.9
Rural		20.1

**Route (to school) characteristics**		
Streetlight density (median (range))	Number of streetlights within 100 m of route divided by route length	9.0 (0–137.7)
Main road en route (%)	Primary (A) road as part of route	
Yes		18.6
Proportion of route within an urban area (%; median (range))	Proportion of route that passes through urban area	100 (0–100)

**School characteristics**		
Travel plan (%)	School has a travel plan (a formal document that identifies ways to encourage walking, cycling, or use of public transport to school)	
Yes		92
Walk to school initiative (%)	The school has a Walk to School initiative (period during which children are encouraged to walk to school)	
Yes		69
Walking accessibility score^d^ (median (range))	Composite measure for accessibility by walking (max=5)	2 (1–4)
Cycling accessibility score^d^ (median (range))	Composite measure for accessibility by cycling (max=7)	4 (1–7)

a The neighborhood comprised the area within an 800 m pedestrian network buffer around the child′s home.

b Seventeen different land uses were classified: farmland, woodland, grassland, uncultivated land, other urban, beach, marshland, sea, small settlement, private gardens, parks, residential, commercial, multiple-use buildings, other buildings, unclassified, buildings, and roads. This score is also known as the Herfindahl–Hirschman index used by Rodriguez and Song (2005).

c Index of multiple deprivation (Department of Communities and Local Government, 2007).

d Refer to Jones et al. (2010).

#### 2.2.4 Perceptions of the social and physical environment

Survey measures of perceptions of the social and physical environment that were significantly associated with active transport to school among these children at T1 (Panter et al., 2010b) were considered and are described in Table 2. Using responses to seven parent′s questionnaire items on perceptions of social cohesion and trust, a sense of community score was computed (internal reliability: Cronbach′s *α*=0.90) (Panter et al., 2010b). Using child questionnaire responses (1 ‘yes’; 0 ‘no’), the following were examined: whether the child was encouraged by a friend or a parent to walk/cycle to school and whether they considered their neighborhood safe for walking or playing alone during the day (Table 2).

**Table 2:** Neighborhood perceptions, social support and parental rules.

	**Description**	**Values**
**Perceptions of neighborhood**		
Sense of community score^a^	Total road lengths divided by neighborhood area	Median 25, range 9–35
Safe to walk or play during the day^b^	Neighborhood is safe to walk or play during the day	Yes 70%
Neighborhood walkability score^a^	Supportiveness of neighborhood area for walking	Median 67, range 35–87
The traffic makes it too dangerous for my child to walk/cycle to school^a^	Parent perceives traffic as a barrier to walking/cycling to school	Agree/strongly agree 17%
I am worried that something will happen to my child on the way to school^a^	Parent is concerned about something happening to child on journey to school	Agree/strongly agree 42%

**Social support**		
Parental encouragement to walk/cycle to school^b^	Parent encourages child to walk/cycle to school	Yes 67%
Peer encouragement to walk/cycle to school^b^	Friend encourages child to walk/cycle to school	Yes 37%

**Parental rules**		
How often does parent restricted their child from walking or cycling to a friend′s house	Frequency of parental restriction of walking/cycling to friend′s house	Often/very often 11%
How often does parent allow child to play^a^ outside anywhere within the neighborhood^a^	Frequency of parent allowing child to play anywhere in n′hood	Often/very often 26%

a Reported by parent.

b Reported by child.

A neighborhood walkability score was computed using responses to 24 items in the parent′s questionnaire on perceptions of how supportive their neighborhood was for walking/cycling. For example, perceptions of road safety, street connectivity, availability of footpaths/bike trails were measured (internal reliability: Cronbach′s *α*=0.74) (Panter et al., 2010a). In addition, parents reported their level of agreement (using a five-point Likert scale) with two items regarding traffic and safety concerns on the route to school (Panter et al., 2010a). Responses were dichotomized: 1 ‘agree/strongly agree’; 0 ‘strongly disagree/disagree/neither’ (Table 2).

#### 2.2.5 Parental rules regarding their child′s physical activity

Parents were asked how often they or their partner (1) restricted their child from walking/cycling to a friend′s house and (2) allowed their child to play outside anywhere within the neighborhood. Responses were dichotomized: 1 ‘often/very often’; 0 ‘else (i.e. never/rarely/sometimes)’ (Table 2).

## 3 Data analyses

In 2013, independent mobility while walking/cycle on the school journey was examined at each time-point. Chi-squared tests of significance were performed to examine significant differences in independent mobility on the school journey by sex of the child. A series of bivariate logistic regression analyses examined cross-sectional associations between each explanatory social and physical environmental variable and the odds of independent mobility on the school journey at T1. Data analyses were conducted in Stata (Stata, version 12.0) using the ‘cluster’ option to allow for non-independence of children within the same school. Analyses were stratified by sex as boys tend to be granted independent mobility at an earlier age than girls (Hillman et al., 1990). All variables that were significantly associated (*p*<0.05) with independent mobility at T1 were entered into a multiple logistic regression model.

To examine social and physical environmental variables at baseline as predictors of change in independent mobility, this analysis strategy was repeated except that the outcome variable was independent mobility on the school journey at T2, controlling for independent mobility at T1. All analyses controlled for parental education level, distance to school and urban/rural location. Between T1 and T2, 20 (4%) children moved home. Analyses were repeated with these children excluded and the final results did not differ substantially. Therefore results of analyses that include data from these children are presented. None of the children moved school.

In these analyses, predictors of independent mobility were examined among children walking and cycling to school as well as those being taken by motorized transport. In order to examine whether these predictors of independent mobility differed in the walking and cycling group compared to the overall sample, regression modeling was repeated amongst this subset of active travelers.

## 4 Results

### 4.1 Sample

Overall, 1121 (54% of the original sample) children aged 9–10 years resided within 1600 m of their school at T1. Almost half (43%) were boys. The median number of siblings was 2 (range 0–8). Parents′ education levels (T1) were spread fairly evenly: low, 28%; medium, 36%; high, 36%. Most households (88%) had access to a car, and over half (54%) had access to multiple cars. Almost all children (94%) owned a bicycle. One-year follow-up data were analyzed for 491 children (44% of the baseline analysis sample). Compared with those who participated at T1 only, the follow-up group had slightly lower proportions of boys (39% vs. 46%, *p*=0.023), higher rates of bike ownership (96% vs. 92%, *p*=0.008) and their households had higher rates of access to multiple cars (57% vs. 51%, *p*=0.020). However, there was no significant difference (*p*=0.657) in parents′ education levels: 37% of parents in the follow-up group reported attaining a high level of (i.e. tertiary) education while 34% of parents who participated only at T1 reported this. Similarly there was no significant difference (*p*=0.069) in the number of siblings: 49% of participants in the follow-up group compared with 55% of those who participated only at baseline had two or more siblings.

### 4.2 Independent mobility on active journeys to school

Travel modes and accompaniment levels on the journey to school are presented in Table 3. At T1 almost three quarters (74%) of those residing within 1600 m of school either walked (62%) or cycled there (12%), but less than half (43%) did so without adult accompaniment. Overall, the rate of independent mobility on active journeys to school increased to 53% at T2, mainly due to increases in walking without adult accompaniment. Compared with girls, a significantly higher (*p*<0.05) proportion of boys walked/cycled independently to school at T1, but at T2 the proportions of boys and girls doing so were not significantly different. Between T1 and T2, 17% of children took up walking/cycling independently to school, while 6% stopped traveling in this way (3% continued to use active transport but did so with adult accompaniment; 3% switched from active transport to car travel). Overall 36% of children walked/cycled independently to school at both time-points, while 40% did so at neither time-point.

**Table 3:** Travel mode and independent mobility among children who reside within 1600 m of their school.

	**All children (%)**	**Boys (%)**	**Girls (%)**
**T1**	*n*=1121	*n*=483	*n*=638
By car	25.2	26.3	24.3
By bus/train	0.4	0.2	0.5
On foot	62.1	55.9	66.8
By bike	12.4	17.6	8.4
**Active independent mobility**	**42.9**	**47.2**	39.8*
On foot (no adult accompaniment)	35.3	34.9	35.7
By bike (no adult accompaniment)	7.6	12.4	4.1

**T2**	*n*=491	*n*=193	*n*=298

By car	18.1	17.1	18.7
By bus/train	0.4	0.5	0.4
On foot	72.8	65.3	77.6
By bike	8.7	17.1	3.3
**Active independent mobility**	**53.4**	**58.0**	**50.4**
On foot (no adult accompaniment)	46.6	43.5	48.7
By bike (no adult accompaniment)	6.7	14.5	1.7

⁎ *p*<0.05 Chi-squared test of significance revealed significant difference by sex.

### 4.3 Cross-sectional associations with independent active transport at T1

The results of logistic regression analyses examining associations between explanatory variables described above and the odds of the child walking/cycling independently to school at T1 are presented in Table 4. Results are presented for the whole sample (i.e. including those who used motorized travel) because in comparison there was little difference in these associations amongst those who walked or cycled to school. More explanatory variables were associated with the odds of boys rather than girls walking/cycling independently to school. However, boys and girls were less likely to walk/cycle independently if their parents were concerned about traffic or worried about something happening to their child on the school journey. In contrast, those with more siblings and whose parents considered it safe to play outside during daytime were more likely to walk/cycle independently. Only one objective measure of the environment, the effective walkable area (four categories based on a quartile split), was associated (negatively) with independent mobility on the school journey at T1, but only for boys. In other words, relative to the lowest category of effective walkable area, each increasing category was associated with reduced odds of boys walking/cycling independently to school at T1. However, boys who were often allowed to play anywhere within their neighborhood were more likely to walk/cycle independently to school at T1, compared with those with more restricted boundaries (Table 4).

**Table 4:** Odds of walking or cycling independently to school at T1.

**Characteristic**	**Boys (*****n*****=483)**		**Girls (*****n*****=638)**	
	**Odds ratio (95% CI)**	**Adjusted odds ratio**^a^**(95% CI)**	**Odds ratio (95% CI)**	**Adjusted odds ratio**^a^**(95% CI)**
***Neighborhood characteristics***				
Road density (quartiles; ref=lowest)	0.84 (0.67, 1.04)	–	0.85 (0.67, 1.09)	–
Proportion of ‘A’ roads (quartiles; ref=lowest)	0.92 (0.70, 1.21)	–	**0.78 (0.63, 0.97)**⁎	0.98 (0.78, 1.22)
Streetlight density (quartiles; ref=lowest)	0.96 (0.75, 1.22)	–	1.00 (0.78, 1.28)	–
Effective walkable area (quartiles; ref=lowest)	**0.77 (0.65, 0.91)**⁎⁎	**0.78 (0.63, 0.96)**⁎	0.86 (0.73, 1.03)	–
Connected node ratio area (quartiles; ref=lowest)	**0.74 (0.62, 0.90)**⁎⁎	0.81 (0.65, 1.01)	**0.72 (0.57, 0.91)**⁎⁎	0.81 (0.63, 1.03)
Junction density (quartiles; ref=lowest)	**0.77 (0.65, 0.92)**⁎⁎	–	0.86 (0.72, 1.02)	–
Land use mix (HHI)^b^ (quartiles; ref=lowest)	1.16 (0.93, 1.44)	–	0.99 (0.78, 1.27)	–
Socioeconomic deprivation (neigh_imd_2007)^c^ (quartiles; ref=lowest)	0.80 (0.64, 1.00)	–	0.88 (0.73, 1.07)	–

***Route (to school) characteristics***				
Streetlight density and route to school (quartiles; ref=lowest)	0.85 (0.69, 1.05)	–	0.97 (0.80, 1.18)	–
Does route to school include a main (‘A’) road (ref=no)				
Yes	0.73 (0.39, 1.35)	–	0.68 (0.38, 1.23)	–
Proportion of route to school within an urban				
Area (quartiles; ref=lowest)	**0.67 (0.50, 0.91)**⁎	0.78 (0.55, 1.12)	0.95 (0.70, 1.28)	–

***School characteristics***				
Does school have a travel plan (ref=no)				
Yes	0.71 (0.41, 1.22)	–	0.97 (0.52, 1.78)	–
Does school have a walk to school initiative (ref=no)				
Yes	0.75 (0.47, 1.19)	–	0.90 (0.58, 1.40)	–
School′s walking access – score (quartiles; ref=lowest)	1.00 (0.72, 1.39)	–	1.13 (0.85, 1.51)	–
School′s cycle access – score (quartiles; ref=lowest)	1.05 (0.84, 1.32)	–	1.02 (0.85, 1.22)	–

***Social Support***				
Friend encouragement for walking/cycling to school (ref=no)				
Yes	**1.88 (1.24, 2.86)**⁎⁎	1.26 (0.81, 1.96)	1.42 (0.98, 2.04)	–
Parent encouragement for walking/cycling to school (ref=no)				
Yes	**1.69 (1.10, 2.59)**⁎	1.54 (0.95, 2.51)	**1.66 (1.10, 2.51)**⁎	1.54 (0.98, 2.40)

***Perceptions of neighborhood***				
Sense of community score (quartiles; ref=lowest)	**1.32 (1.10, 1.58)**⁎⁎	1.23 (0.99, 1.53)	1.19 (0.99, 1.42)	–
Neighborhood is safe place for walking or playing during the day (ref=no)				
Yes	**2.67 (1.74, 4.09)**⁎⁎⁎	**2.20 (1.31, 3.68)**⁎⁎	**1.98 (1.34, 2.92)**⁎⁎	**1.71 (1.07, 2.73)**⁎
Neighborhood walkability score (quartiles; ref=lowest)	**1.25 (1.02, 1.54)**⁎	1.02 (0.81, 1.29)	**1.23 (1.05, 1.44)**⁎	1.10 (0.90, 1.36)
The traffic makes it too dangerous for my child to walk or cycle to school (ref=do not agree)				
Agree (or strongly agree)	**0.13 (0.06, 0.30)**⁎⁎⁎	**0.19 (0.07, 0.55)**⁎⁎	**0.21 (0.13, 0.35)**⁎⁎⁎	**0.37 (0.21, 0.65)**⁎⁎⁎
I am worried that something will happen to my child on the way to school (ref=do not agree)				
Agree (or strongly agree)	**0.37 (0.24, 0.58)**⁎⁎⁎	**0.59 (0.36, 0.96)**⁎	**0.36 (0.24, 0.55)**⁎⁎⁎	**0.47 (0.29, 0.75)**⁎⁎

***Parental rules***				
How often do you or your partner restrict your child from walking/cycling to a friend′s house? (ref=not often)				
Often (or very often)	**0.45 (0.22, 0.92)**⁎	0.72 (0.29, 1.81)	0.59 (0.31, 1.11)	–
How often do you or your partner allow your child to play outside anywhere within the neighborhood (ref=not often)				
Often (or very often)	**2.88 (1.80, 4.61)**⁎⁎⁎	**1.80 (1.02, 3.17)**⁎	**2.35 (1.54, 3.61)**⁎⁎⁎	1.70 (0.99, 2.92)

***Household/family characteristics***				
Number of siblings (continuous)	**1.14 (1.01, 1.28)**⁎	**1.20 (1.03, 1.42)**⁎	**1.19 (1.04, 1.36)**⁎	**1.20 (1.03, 1.40)**⁎
Car (ref=no)				
Yes	0.93 (0.50, 1.72)	–	0.67 (0.41, 1.10)	–
Multiple cars (ref=no)				
Yes	0.67 (0.44, 1.02)	–	1.14 (0.77, 1.70)	–

All analyses controlled for parental education level, distance from home to school and urban/rural status.

–: Not significant.

a All variables that were found to be significantly associated (*p*<0.05) with independent mobility at T1 (except ‘junction density’) were entered together into a multiple logistic regression model. Collinearity was detected between ‘effective walkable area’ and ‘junction density’ (VIF>295). The variable ‘effective walkable area’ was more strongly associated with the dependent variable and was retained.

⁎ *p*<0.05.

⁎⁎ *p*<0.01.

⁎⁎⁎ *p*<0.001.

b Seventeen different land uses were classified: farmland, woodland, grassland, uncultivated land, other urban, beach, marshland, sea, small settlement, private gardens, parks, residential, commercial, multiple-use buildings, other buildings, unclassified buildings, and roads. This score is also known as the Herfindahl–Hirschman index used by Rodriguez and Song (2005).

c Index of multiple deprivation (Department of Communities and Local Government, 2007).

### 4.4 Prospective associations with change in independent active transport between T1 and T2

At T2, in the multivariable regression model (Table 5) only one baseline variable was associated longitudinally with increased odds of boys walking/cycling independently to school: parents often allowing their child to play outside anywhere within the neighborhood; and one further variable, household access to a car, was associated with decreased odds of this. Only one variable, land use mix, was positively associated longitudinally with girls walking/cycling independently to school, while two further variables were negatively associated with girls doing so: the proportion of main roads in the neighborhood; and parental encouragement for walking/cycling to school (Table 5).

**Table 5:** Odds of walking or cycling independently to school at T2 (controlling for doing so at T1).

**Characteristic**	**Boys (*****n*****=193)**		**Girls (*****n*****=298)**	
	**Odds ratio (95% CI)**	**Adjusted odds ratio**^a^**(95% CI)**	**Odds ratio (95% CI)**	**Adjusted odds ratio**^a^**(95% CI)**
***Neighborhood characteristics***				
Road density (quartiles; ref=lowest)	0.91 (0.65, 1.28)	–	1.15 (0.87, 1.53)	–
Proportion of ‘A’ roads (quartiles; ref=lowest)	0.90 (0.60, 1.34)	–	**0.65 (0.48, 0.87)**⁎⁎	**0.67 (0.47, 0.94)**⁎
Streetlight density (quartiles; ref=lowest)	0.91 (0.62, 1.33)	–	1.07 (0.81, 1.43)	–
Effective walkable area (quartiles; ref=lowest)	1.12 (0.77, 1.64)	–	0.82 (0.63, 1.07)	–
Connected node ratio area (quartiles; ref=lowest)	0.84 (0.64, 1.10)	–	1.11 (0.88, 1.38)	–
Land use mix (HHI)^b^ (quartiles; ref=lowest)	0.99 (0.69, 1.42)	–	**1.32 (1.04, 1.67)**⁎	**1.38 (1.06, 1.79)**⁎
Socioeconomic deprivation (neigh_imd_2007)^c^ (quartiles; ref=lowest)	1.25 (0.87, 1.80)	–	0.91 (0.73, 1.12)	–

***Route to school characteristics***				
Streetlight density and route to school (quartiles; ref=lowest)	0.94 (0.75, 1.18)	–	1.18 (1.00, 1.40)	–
Does route to school include a main (‘A’) road (ref=no)				
Yes	1.20 (0.42, 3.39)	–	0.58 (0.32, 1.05)	–
Proportion of route to school within an urban				
Area (quartiles; ref=lowest)	1.11 (0.66, 1.86)	–	0.81 (0.53, 1.22)	–
Does school have a travel plan (ref=no)				
Yes	1.53 (0.87, 2.69)	–	1.05 (0.70, 1.58)	–
Does school have a walk to School initiative (ref=no)				
Yes	1.49 (0.69, 3.23)	–	1.05 (0.57, 1.95)	–
School′s walking access – score (quartiles; ref=lowest)	1.22 (0.82, 1.83)	–	0.90 (0.64, 1.25)	–
School′s cycle access – score (quartiles; ref=lowest)	1.01 (0.75, 1.34)	–	1.00 (0.80, 1.25)	–

***Social support***				
Friend encouragement for walking/cycling to school (ref=no)				
Yes	1.00 (0.51, 1.93)		1.15 (0.66, 2.00)	–
Parent encouragement for walking/cycling to school (ref=no)				
Yes	1.16 (0.57, 2.34)	–	**0.43 (0.23, 0.83)**⁎	**0.40 (0.20, 0.80)**⁎⁎

***Perceptions of neighborhood***				
Sense of community score (quartiles; ref=lowest)	1.09 (0.75, 1.58)	–	0.83 (0.65, 1.07)	–
Neighborhood is safe place for walking or playing during the day (ref=no)				
Yes	1.06 (0.49, 2.31)	–	**2.12 (1.07, 4.20)**⁎	1.98 (0.97, 4.05)
Neighborhood walkability score (quartiles; ref=lowest)	1.29 (0.88, 1.90)	–	1.04 (0.82, 1.33)	–
The traffic makes it too dangerous for my child to walk or cycle to school (ref=do not agree)				
Agree (or strongly agree)	0.45 (0.13, 1.55)	–	0.58 (0.29, 1.18)	–
I am worried that something will happen to my child on the way to school (ref=do not agree)				
Agree (or strongly agree)	0.62 (0.27, 1.40)	–	0.85 (0.44, 1.63)	–

***Parental rules***				
How often do you or your partner restrict your child from walking/cycling to a friend′s house? (ref=not often)				
Often (or very often)	0.48 (0.16, 1.45)	–	0.76 (0.34, 1.71)	–
How often do you or your partner allow your child to play outside anywhere within the neighborhood (ref=not often)				
Often (or very often)	**3.40 (1.34, 8.66)**⁎	**3.14 (1.24, 7.96)**⁎	1.24 (0.62, 2.48)	–

***Household/family characteristics***				
Number of siblings (continuous)	0.97 (0.70, 1.33)	–	1.04 (0.87, 1.26)	–
Car (ref=no)				
Yes	**0.22 (0.06, 0.87)**⁎	**0.27 (0.08, 0.94)**⁎	0.98 (0.46, 2.06)	–
Multiple cars (ref=no)				
Yes	0.76 (0.36, 1.57)	–	1.49 (0.83, 2.66)	–

All analyses controlled for parental education level, distance from home to school and urban/rural status.

–: Not significant.

a All variables that were found to be significantly associated (*p*<0.05) with independent mobility at T2 were entered together into a multiple logistic regression model.

⁎ *p*<0.05.

⁎⁎ *p*<0.01.

b Seventeen different land uses were classified: farmland, woodland, grassland, uncultivated land, other urban, beach, marshland, sea, small settlement, private gardens, parks, residential, commercial, multiple-use buildings, other buildings, unclassified buildings, and roads. This score is also known as the Herfindahl–Hirschman index used by Rodriguez and Song (2005).

c Index of multiple deprivation (Department of Communities and Local Government, 2007).

## 5 Discussion

Despite growing interest in children′s independent mobility and its positive associations with physical activity (Schoeppe et al., 2013), limited research exists on social and physical environmental factors that may influence children′s independent mobility to school. In particular, most related studies are cross-sectional, thus precluding causal inference. For example, *a* Taiwanese study reported that density of trees providing shade and prevalence of footpaths were associated with children walking to school without adult accompaniment, while intersection density was inversely associated with this (Lin and Chang, 2010). Most studies of environmental factors related to children′s school journeys have examined travel mode (e.g. active transport vs. motorized modes) rather than whether the child walked/cycled *independently* (Mitra, 2013).

In literature on children′s independent mobility, the parent is considered literally as a ‘gatekeeper’ who controls their children′s mobility outside the home through the granting of ‘mobility licenses’ (e.g. allowing them to cross roads alone) (Hillman et al., 1990). While interest has grown in aspects of the parent–child interaction that determine whether the school journey is made independently, pathways that lead to independent mobility on the school journey are under-researched (Mitra, 2013). A framework proposed recently by Mitra (2013) posits that family members determine the travel mode to school and whether the journey is made independently based on: perceived importance of escorting the child; mobility choices (e.g. car access) and restrictions (e.g. the parent′s work commute). Furthermore the framework identifies five domains of influence on travel mode and on independent mobility to school: (1) distance to school; (2) safety concerns (traffic; personal); (3) street connectivity; (4) pedestrian facilities and esthetics; (5) social connectedness. While the current study does not directly test Mitra′s framework (2013), aspects of all domains are examined in relation to children′s independent mobility to school.

This is among the first longitudinal studies to examine social and physical environmental factors that may be associated with changes in children′s independent mobility to school. Despite residing within an appropriate walking distance (Timperio et al., 2004), less than half of the children walked/cycled independently to school. Consistent with earlier research (Hillman et al., 1990, Prezza et al., 2001) boys compared with girls had higher rates of independent mobility but this difference narrowed as they grew older. Baseline findings demonstrate that, overall, social environmental variables and perceptions of the neighborhood were associated more frequently with children walking/cycling to school without adult accompaniment than were objective measures of the physical environment. However, the discussion shall focus on the novel longitudinal component of this study.

Only one variable was associated longitudinally with increased odds of boys′ walking/cycling independently to school: being allowed by their parents to play outside within their neighborhood more often. This variable was also associated cross-sectionally with boys′ independent mobility to school and may be indicative of boys′ broader independent mobility at an earlier age than girls (Hillman et al., 1990, Prezza et al., 2001). For girls, greater land use mix in the neighborhood (suggesting co-location of residences, business/retail outlets, sports facilities) was associated longitudinally with increased odds of walking/cycling independently to school. While the location of walkable destinations near home has been shown to promote active transport among adolescents, the association with children′s independent mobility is less clear (Giles-Corti et al., 2009).

For girls, the proportion of main roads within their neighborhood was associated longitudinally with reduced odds of walking/cycling independently to school. Perceptions of busy roads may have contributed to concern about traffic which is a key barrier to children′s independent active transport (Carver et al., 2008). There is evidence that from early childhood, girls are encouraged by their parents to take fewer risks than are boys (Morrongiello and Dawber, 1999), so traffic concerns may provide a greater deterrent to girls′ walking/cycling. Parental encouragement of girls′ walking/cycling to school was negatively associated with doing so independently. While this association was not in the expected direction, it is possible that parents who were concerned about their daughters′ inactivity were encouraging them to walk/cycle more, and in some cases accompanied them. For example, among 35 girls who were encouraged by their parents to walk/cycle to school but traveled there by car at T1, 14 had switched to walking at T2: eight were accompanied by a parent while six walked independently. Encouragement of girls′ physical activity as they become older is of great importance as age-related declines in adolescent girls′ physical activity levels are well-documented (Kimm et al., 2005, Van Mechelen, 2000).

It should be noted that since features of the objective physical environment were not measured at T2 it was not possible to assess whether the environment had changed between T1 and T2. Any changes may have contributed to change in independent mobility to school. However, our sample was drawn from a large geographic area, and substantial changes in the physical environment (e.g. to the road network or land use mix) will not be common over a short follow up period (12 months). They are hence unlikely to explain much of the change in independent mobility in this study. It may be however that parents′ perceptions of the environment and demographic characteristics such as household car access, which were not re-measured at T2, may change over a short time, and this is recognized as a limitation.

In these analyses, predictors of independent mobility were examined among children who used active transport to school as well as those who used motorized transport. In the case of children currently being driven, interventions to increase the prevalence of independent mobility on the school commute will need to achieve modal shift to an active form of transport in addition to a switch from an accompanied to unaccompanied journey. Compared with results for the whole sample presented in this paper, few differences were found in how characteristics of the neighborhood, school and route to school were associated with independent travel amongst these walkers and cyclists. The main differences were that perceptions of the neighborhood and social support were no longer associated with the odds of independent mobility. Since these variables were shown previously to be associated with travel mode to school (Panter et al., 2010b), this is unsurprising.

A major strength of this study is the inclusion of data gathered using seasonally-matched surveys of parents and their children attending primary schools in urban and rural areas. However, recruitment of children from one county in England during one season may limit the generalizability of findings nationally and internationally. While the low retention rate and narrow age-range of participating children, reliance on their self-report and assumption of the routes to school (which may not correspond to actual routes taken) are acknowledged as limitations, a further strength is the inclusion of objective measures and perceptions of the environment in the child′s neighborhood, on the route to school and in the school environment.

## 6 Conclusions

Despite relatively high rates of walking and cycling (Carver et al., 2012, McDonald, 2007) only around half of the children walked/cycled without adult accompaniment. Interventions increasing independent mobility are required. As well as offering potential to increase children′s physical activity and provide related health benefits through active transport, such interventions can also promote children′s social, cognitive and emotional development via their walking/cycling without adult accompaniment (Kyttä, 2004). These findings suggest that it may be worthwhile for interventions to develop parents′ skills in order to teach their children to be independently mobile and to build parents′ and children′s confidence to venture out without parental accompaniment. In addition, urban planners should consider designing neighborhoods in which residences, business/retail outlets and sports facilities are co-located to promote active transport among youth, as well as among the broader population (National Institute for Health and Clinical Excellence, 2013).
